# CRISPR/Cas9: a powerful genetic engineering tool for establishing large animal models of neurodegenerative diseases

**DOI:** 10.1186/s13024-015-0031-x

**Published:** 2015-08-04

**Authors:** Zhuchi Tu, Weili Yang, Sen Yan, Xiangyu Guo, Xiao-Jiang Li

**Affiliations:** Institute of Genetics and Developmental Biology, Chinese Academy of Sciences, Beijing, 100101 China; Department of Human Genetics, Emory University School of Medicine, Atlanta, GA 30322 USA

**Keywords:** CRISPR/Cas9, Non-human primates, Neurodegenerative diseases, Animal model

## Abstract

Animal models are extremely valuable to help us understand the pathogenesis of neurodegenerative disorders and to find treatments for them. Since large animals are more like humans than rodents, they make good models to identify the important pathological events that may be seen in humans but not in small animals; large animals are also very important for validating effective treatments or confirming therapeutic targets. Due to the lack of embryonic stem cell lines from large animals, it has been difficult to use traditional gene targeting technology to establish large animal models of neurodegenerative diseases. Recently, CRISPR/Cas9 was used successfully to genetically modify genomes in various species. Here we discuss the use of CRISPR/Cas9 technology to establish large animal models that can more faithfully mimic human neurodegenerative diseases.

Neurodegenerative diseases — Alzheimer’s disease(AD),Parkinson’s disease(PD), amyotrophic lateral sclerosis (ALS), Huntington’s disease (HD), and frontotemporal dementia (FTD) — are characterized by age-dependent and selective neurodegeneration. As the life expectancy of humans lengthens, there is a greater prevalence of these neurodegenerative diseases; however, the pathogenesis of most of these neurodegenerative diseases remain unclear, and we lack effective treatments for these important brain disorders.

## Genetic rodent models of neurodegenerative diseases

Animal models provide us with a valuable system for the study of neurodegenerative diseases. Transgenic mouse models are particularly useful, since we have versatile genetic tools available to modify the genomes of mice to create loss- or gain-of-function models. Most neurodegenerative diseases, such as AD, PD, and ALS, are sporadic, and only a small percentage of cases (4-5 %) are caused by genetic mutations [1–5]. However, HD is a monogenic mutation disease caused by CAG repeat expansions in the IT15 gene, resulting in expanded polyglutamine repeats in huntingtin (htt) [6, 7]. Identification of the genetic mutations for different neurodegenerative diseases has enabled the generation of a variety of transgenic mouse models via expression of mutant proteins. As a result, we now have various mouse models of neurodegenerative diseases from the expression of mutant genes under different promoters or from using other transgenic approaches.

There are a number of excellent reviews covering different types of neurodegenerative diseases and their genetic mouse models [8–12]. Investigations of different mouse models of neurodegenerative diseases have revealed a common pathology shared by these diseases. First, the development of neuropathology and neurological symptoms in genetic mouse models of neurodegenerative diseases is age dependent and progressive. Second, all the mouse models show an accumulation of misfolded or aggregated proteins resulting from the expression of mutant genes. Third, despite the widespread expression of mutant proteins throughout the body and brain, neuronal function appears to be selectively or preferentially affected. All these facts indicate that mouse models of neurodegenerative diseases recapitulate important pathologic features also seen in patients with neurodegenerative diseases.

However, it seems that mouse models can not recapitulate the full range of neuropathology seen in patients with neurodegenerative diseases. Overt neurodegeneration, which is the most important pathological feature in patient brains, is absent in genetic rodent models of AD, PD, and HD. Many rodent models that express transgenic mutant proteins under the control of different promoters do not replicate overt neurodegeneration, which is likely due to their short life spans and the different aging processes of small animals. Also important are the remarkable differences in brain development between rodents and primates. For example, the mouse brain takes 21 days to fully develop, whereas the formation of primate brains requires more than 150 days [13]. The rapid development of the brain in rodents may render neuronal cells resistant to misfolded protein-mediated neurodegeneration. Another difficulty in using rodent models is how to analyze cognitive and emotional abnormalities, which are the early symptoms of most neurodegenerative diseases in humans. Differences in neuronal circuitry, anatomy, and physiology between rodent and primate brains may also account for the behavioral differences between rodent and primate models.

### Current large animal models of neurodegenerative diseases

Several species have been used to create large animal models of neurodegenerative diseases. Of these, pigs, sheep, and non-human primates have been used successfully to establish HD, ALS, and PD animal models.

HD is caused by the expansion of a polyglutamine (polyQ) repeat (>37 glutamines) in the N-terminal region of the disease protein huntingtin (htt) and is characterized by preferential neuronal loss in distinct brain regions [6, 14]. Numerous mouse models of HD have been investigated extensively, revealing that N-terminal fragments of mutant htt with expanded polyQ repeats are toxic and form aggregates or inclusions in the brain [15–24]. Transgenic HD pigs that express N-terminal mutant htt consisting of the first 208 amino acids with 105Q (N208-105Q) were generated using nuclear transfer technology [25]. Primary porcine fetal fibroblast cells expressing N-terminal mutant htt fragments were used for the nuclear transfer, and pig eggs containing these nuclei were developed to early embryos that were then transferred to surrogate pigs to produce newborn pigs. Due to the overexpression of toxic N-terminal mutant htt, most of the transgenic HD piglets died postnatally, and some of them showed a severe chorea phenotype before death. However, transgenic mice expressing the same mutant htt could live up to 9 months, suggesting that mutant htt is more toxic to larger animals [25]. More importantly, all transgenic pig brains had obvious apoptotic cells, which have not been seen in the brains of many HD mouse models. Interestingly, apoptotic cardiomyocyte loss occurs in the absence of mutant htt aggregates in cardiac tissue in R6/2 mice, suggesting that this peripheral apoptotic event can be driven by mutant htt-mediated CNS dysfunction [26]. In other experiments, transgenic pigs expressing large htt fragments with shorter polyQ expansion showed no obvious neurological phenotypes, supporting the idea that shorter htt fragments with larger polyQ expansions are more toxic [27, 28]. Similarly, Jacobsen and colleagues expressed transgenic full-length human htt cDNA containing 73 CAG repeats in sheep under the control of the human promoter by microinjection. One of the founders showed robust expression of the full-length human htt protein in both CNS and non-CNS tissue. Although there was decreased expression of the neuronal marker DARPP-32 in medium-sized spiny neurons in the striatum at 7 months, these full-length HD sheep grow normally [29]. Thus, differences between full-length and N-terminal htt transgenic large animals provide further evidence for the toxicity of N-terminal mutant htt. Because of the gain-of-toxic function in HD, current therapeutic approaches have been focused on lowing mutant htt expression, including siRNA, anti-sense oligonuelotides [30] as well as Zinc Finger protein strategies [31]. These approaches, however, are mainly applied to rodent models of HD. Thus, the development of larger animal models might provide a useful tool to validate the efficacy of ongoing pre-clinical trials in rodents.

Amyotrophic lateral sclerosis (ALS) is an adult-onset, progressive neurodegenerative disease characterized by the selective death of motor neurons in the motor cortex, brainstem, and spinal cord [32–34]. Most ALS patients suffer from the sporadic form of ALS, with the other 5 %-10 % of patients presenting with familial ALS. Familial ALS could be caused by mutations in one of at least 32 known genetic loci, including superoxide dismutase 1 (SOD1), TAR DNA-binding protein 43 (TDP-43), fused in sarcoma (FUS), and C9ORF72 [35–38]. The nuclear transfer method has also been used to establish cloned pigs expressing mutant SOD1 protein with the ALS-associated SOD1 mutation G93A [39]. Transgenic SOD1 pigs show germline-transmissible motor defects as well as neuronal degeneration that are dose and age-dependent. More importantly, in the early disease stage, mutant SOD1 did not form cytoplasmic inclusions, but showed nuclear accumulation and ubiquitinated nuclear aggregates, which are seen in some ALS patients, but not in transgenic ALS mouse models [40–42]. This difference between transgenic ALS pigs and mice lends further support to the idea that pig models can mimic some pathological events that occur in patients, but not in mice.

It seems that aging is necessary for AD transgenic pigs to develop impaired memory when the APPsw transgene is expressed, since objective recognition was found to be no different between AD transgenic pigs and controls at 1–2 years of age [43]. It is also possible that high expression levels of transgenic mutant protein are required to facilitate disease progression in large animals.

Non-human primates would be a better model than other animals to mimic the cognitive and emotional abnormalities seen in patients with neurodegenerative diseases. Creation of the first transgenic monkey in 2001 demonstrated that the monkey genome could be genetically modified [44–46]. Later, Yang et al. generated a transgenic HD rhesus monkeys by injecting lentiviral vector into fertilized oocytes to express exon1 mutant htt with 84 CAG repeats [47]. Consistent with the pathology seen in HD mouse models and patients, transgenic HD monkey brains also show abundant htt protein aggregates in neuronal nuclei and neuronal processes. However, unlike HD transgenic mice that express the same exon1 mutant htt, HD transgenic monkeys experience mutant htt level-dependent postnatal death [47]. Furthermore, some HD monkeys develop key clinical features of HD, including dystonia, chorea, and seizure, which can not be found in mouse models or other small animal models. More importantly, HD monkeys display degeneration of axons and neuronal processes without obvious cell body degeneration [25], suggesting that neuronal degeneration in HD may initiate from neuronal processes.

Transgenic monkeys that express mutant α-synuclein to model Parkinson’s disease (PD) have also been established recently [48]. PD is an age-dependent neurodegenerative disease with late-onset degeneration of dopaminergic neurons in the substantia nigra, which leads to a complex motor disorder characterized by bradykinesia, tremor, rigidity, and postural instability. Lewy body inclusions, which are composed mainly of α-synuclein and ubiquitin, and selective loss of dopamine (DA) neurons in the substantia nigra pars compacta are the pathologic and anatomical hallmarks of PD [49–51]. Rodent and other small animal models replicate the pathological and clinical features of human Parkinsonism only partially [52, 53]. For example, most transgenic PD mice show no loss of substantia nigra dopaminergic neurons [54]. By expressing mutant α-synuclein(A53T)in transgenic rhesus monkeys via lentiviral vector expressing A53T in fertilized monkey embryos, we obtained six transgenic A53T monkeys. After the age of 2.5 years, the oldest transgenic A53T monkey started to show age-dependent non-motor symptoms, including cognitive defects, an anxiety phenotype, and poor fine finger coordination and dexterity [48]. These behavioral phenotypes of the A53T monkey are consistent with the non-motor symptoms of PD patients at the early disease stage [48, 51, 55]. The transgenic A53T monkeys demonstrate the age-dependent non-motor symptoms caused by mutant α-synuclein and offer insight into treatment for early PD. Consistent with the age-dependent neuropathology seen in PD, stereotaxic injection of lentiviral vectors expressing A53T in the substantial nigra of monkeys at different ages also revealed that aging promotes neuropathology in non-human primate brains [56]. Transgenic large animal models thus provide us with valuable information about disease pathogenesis and neuropathology that may not be identified in rodent or small animal models.

### CRISPR/Cas9 as a new tool for generating large animal models of neurodegenerative diseases

The previously established large animal models of neurodegenerative diseases have been used to mimic gains of toxic function of mutant proteins. This is because the transgenic approaches used allow for the expression of extra copies of mutant genes under the exogenous promoters; however, many human diseases, including neurodegenerative disorders, are caused by genetic mutations in endogenous genes. Due to the lack of embryonic stem cell lines from large animals for genomic manipulation, it has been difficult to create large animal disease models by genetically modifying endogenous genes. Fortunately, recent developments in genome editing with new technologies now make it possible to establish large animal models to investigate neurodegenerative diseases.

**Fig. 1:**
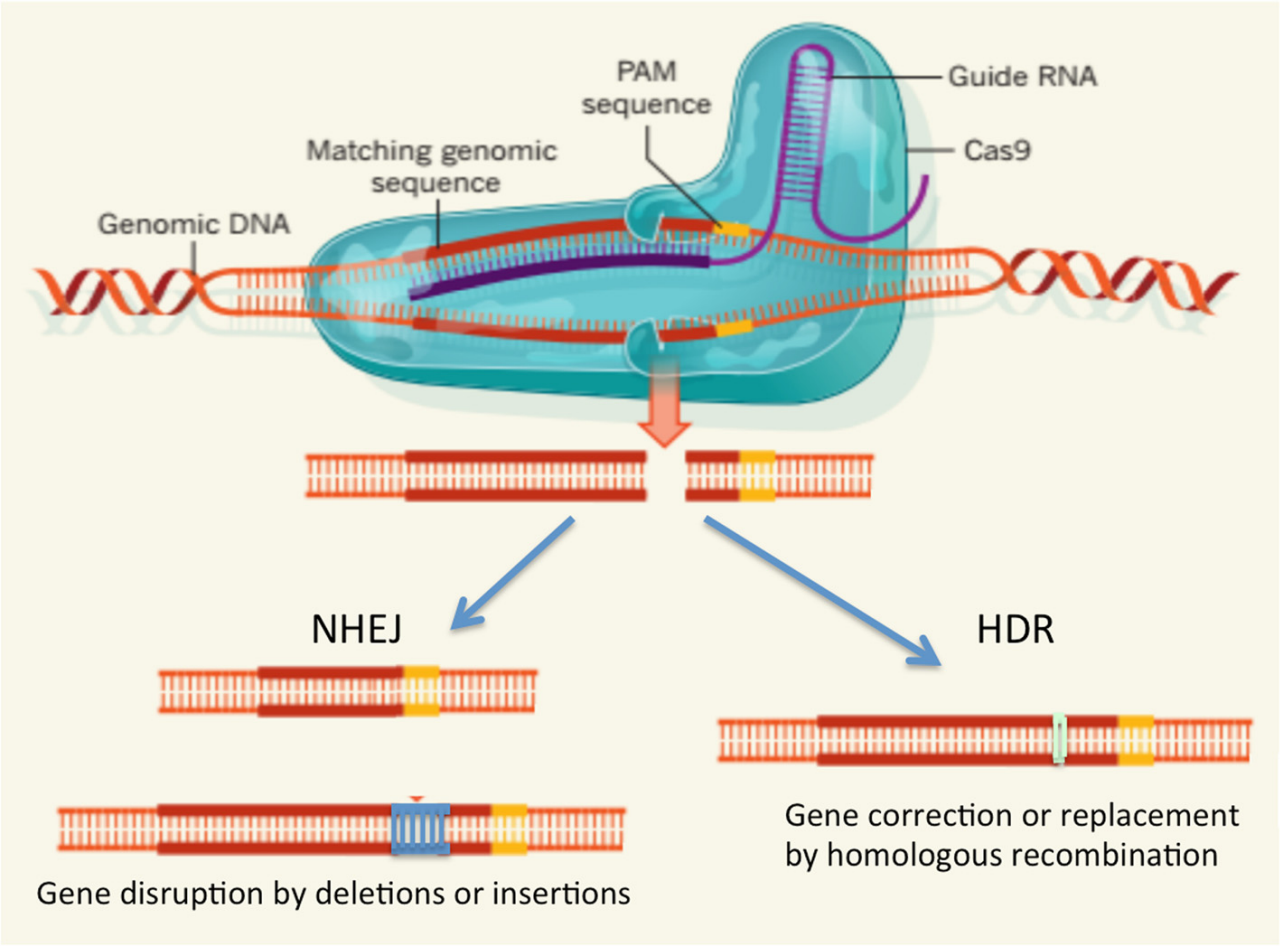
CRISPR-Cas9 targeting system. In the CRISPR/Cas9 system, a guide RNA hybridizes a 20-nt DNA sequence immediately preceding an NGG DNA motif (protospacer-associated motif or PAM), resulting in a double-strand break (DSB) 3 bp upstream of the NGG. The double-stranded DNA breaks become substrates for endogenous cellular DNA repair machinery that catalyze nonhomologous end joining (NHEJ) or homology-directed repair (HDR). Adopted from Charpentier & Doudna, Nature, 2013,495:50–1

The CRISPR(clustered regularly interspaced short palindromic repeats)/Cas9 system is a novel genome modification method in which guide RNAs (gRNA)direct the nuclease Cas9 to selected sequences of genomic DNA, and Cas9 cuts both strands at a precise location. The genomic DNA is then repaired by non-homologous end joining (NHEJ) or homology-directed repair (HDR), resulting in mutations that can interrupt the open reading frame and cause gene inactivation. To simplify the process of constructing the CRISPR/Cas9 system and to keep its cleavage efficacy, the dual-crRNA:tracrRNA complex was designed as a single transcript (single-guide RNA or sgRNA) that is required for Cas9’s binding and cutting DNA targets to introduce double-strand breaks (DSBs) (Fig. 1). CRISPR/Cas9 has now become a simple and versatile RNA-directed system for genome editing in a wide range of different organisms and cell types, including bacteria, mice, rat, zebrafish, pig, human somatic cells, and human pluripotent stem cells [57–61]. Several groups in China recently used CRISPR/Cas9 to genetically modify genomes in embryos from pigs [62, 63] and monkeys [64–67]. These studies proved that CRISPR/Cas9 is an efficient tool to genetically modify genomic DNAs in germline cells without the need to establish embryonic stem cells for genomic manipulation.

Transcription activator-like effector nucleases (TALENs) have also been recently used for genomic editing. TALENs are chimeric proteins consisting of a programmable DNA-binding domain fused to the Fok1 nuclease domain, allowing for cutting double strand DNAs at any desired sequences [68, 69]. Because the binding of TALEN to the targeted DNA relies on its DNA binding domain that is composed of multiple repeat units, each repeat containing 33–35 amino acids, TALENs is thought to have less off-target effect [70]. However, assembling TALEN targeting vectors requires much more efforts than generating CRISPR/Cas9 targeting vectors, making CRISPR/Cas9 a more widely used tool for genomic editing. In this review, we focus on the utilization of CRISPR/Cas9 in establishing large animal models of neurodegenerative diseases.

### Advantages and limitations of CRISPR/Cas9 in large animals

One advantage of CRISPR/Cas9 is that it can create mutations at virtually any location in genomic DNA [60, 71–73]. In the CRISPR/Cas9 system, a guide RNA hybridizes a 20-nt DNA sequence immediately preceding an NGG DNA motif (protospacer-associated motif or PAM), resulting in a double-strand break (DSB) 3 bp upstream of the NGG (Fig. 1). Because this targeting relies on 23 base pair matches, CRISPR/Cas9 can target to virtually any genes in a sequence-dependent manner. As a result, CRISPR/Cas9 can target two alleles to cause a null mutation in the founder animals. Such an advantage is particularly important for generating large animal models of diseases since the extended time to breed large animals does not allow for quick mating of heterozygous mutant animals to generate homozygous mutants. In addition, since CRISPR/Cas9 can disrupt two alleles in female animals, the female animals can also show phenotypes due to the complete loss of function of the targeted gene, even if the disease gene is X-linked inherited. To ensure that two alleles are disrupted, multiple sites of the desired gene can be targeted by CRISPR/Cas9. In this regard, CRISPR/Cas9 makes a powerful tool for generating non-human primate or large animal models of neurodegenerative diseases that are caused by the loss of function of specific genes.

However, because targeting by CRISPR/Cas9 relies on approximate 23 base pair matches [74], CRISPR/Cas9 may generate a number of nonspecific mutations in the genome. Such nonspecific mutations can be diluted over generations in small animals with short breeding times, but for large animals like monkeys, their sexual maturation usually requires 4–5 years; thus the off-target issue is critical in large animals and can confound the phenotypes of founder animals caused by targeting the desired gene.

The second issue with CRISPR/Cas9 is mosaic mutations, or different types of mutations generated in different cells. Mosaic mutations may affect the generation of animal models of genetic human diseases because a specific genetic mutation in a human disease often occurs at the one-cell stage before cell division, such that the same mutation is present ubiquitously in every individual cell. The mechanism behind these mosaic mutations remains unknown. It is possible that the translation of Cas9 mRNA to produce an active enzymatic form is delayed until after the first cell division, and this delay may play a major role in genetic mosaicism [74–77]. The mosaicism problem may also result from the prolonged expression of Cas9 mRNA. Alternatively, differential DNA repair and non-homozygous recombination activities in zygotes and divided embryonic cells can also influence genetic mutation rates and mosaicism. Despite the unknown mechanisms behind mosaicism, mosaic mutations can result in loss of function if they disrupt the expression of functional proteins. Evidence for this comes from a Duchenne muscular dystrophy (DMD) monkey model [65], in which three different mutations in the dystrophin gene cause the loss of dystrophin in monkey muscle and muscle atrophy, as seen in DMD patients [78, 79]. Thus, if mosaic mutations in large animals result in loss of function, the mutant animals may show pathology similar to that caused by a monogenic mutation in human diseases.

Although CRISPR/Cas9 has been used to generate knock-in mutations in various species, this knock-in rate is much lower than the random indel mutation rate because the knock-in requires the precise homologous recombination of the donor DNA. Thus, the current application of CRISPR/Cas9 is used mainly to create random mutations in the targeted genes to induce loss-of-function phenotypes in animal models.

### Use of CRSPR/Cas9 to generate large animal models of neurodegenerative diseases

The ability of CRISPR/Cas9 to directly target any gene in the embryo genome opens up a new avenue for us to generate animal models of neurodegenerative diseases, especially large animal models that often require the investigation of founder animals. As we discussed above, CRISPR/Cas9 can cause mutations in one or two alleles, which can mimic heterozygous or homozygous knockout of a specific gene. Some neurodegenerative diseases, such as PD, can be caused by loss of function due to mutations in the Parkin and Pink1 genes. These genes can be targeted by CRISPR/Cas9 in non-human primates or other large animals to inactivate gene expression. When both alleles are mutated, the complete loss of Parkin or Pink1 will mimic the genetic mutations in PD patients. To ensure that two alleles will be disrupted, multiple targeting regions can be designed, with a few gRNAs for co-injection with Cas9 into fertilized eggs at the one-cell stage.

Many neurodegenerative diseases are also caused by a gain of toxicity of mutant proteins. For example, PD can be caused by mutations in α-synuclein, and HD is caused by polyQ expansion in htt. To generate animal models of such diseases will require knock-in mutations in the genes encoding for the disease proteins. Although the current knock-in rate with CRISPR/Cas9 is low, rapidly developing technology has improved its knock-in efficiency. For example, the use of inhibitors of NHEJ significantly increased the knock-in rate in mammalian cells [80]. Recently, direct nuclear delivery of Cas9 protein complex with chemically synthesized dual RNA was reported to generate knock-in mice with up to 50 % efficiency [81]. Thus, the newly developed CRISPR/Cas9 system holds great promise for use in non-human primates and large animals to generate knock-in models of human diseases or to modify specific genes.

The off-target and mosaic issues in large animal models need to be considered carefully. This is because large animals like monkeys need to be analyzed before producing offspring; thus, the off-target issue is critical and can confound the phenotypes of founder animals. Because off-target events were not seen in established CRISPR/Cas9-targeted monkeys [40–42], it is likely that any off-target effect is quite minimal and can be prevented by designing highly selective gRNA containing adequate mismatched base pairs with other genes. Use of bioinformatic screening to search for unique genomic targets and use of paired Cas9 nickases can also reduce off-targets [59, 82]. Because promotion of HDR over NHEJ has been found to increase knock-in targeting [80, 83], drugs or chemicals that are able to increase this promotion should also help decrease the frequency of off-targets and increase knock-in rate.

If we want to create animal models with loss-of-function phenotypes, mosaic mutations can achieve this goal by disrupting gene function. It is important, however, to ensure that the targeted gene has lost its function. We have recently shown that indel mutations in more than 87 % of the monkey dystrophin gene is sufficient to lead to the loss of expression of dystrophin and consequently results in muscle degeneration as seen in Duchenne muscular dystrophy (DMD) [65]. Thus, quantification of the rates of mosaic mutations, especially those that can disrupt gene expression, is important to assess the functional inactivation of the targeted gene.

Given that DNA repair mechanisms may not be identical in germline and postmitotic neuronal cells, it remains unclear whether there are lower mosaic mutation rates in mature neuronal cells in adult animals. Since CRISPR/Cas9 can also target genes in adult neuronal cells [84–86], it can be applied to the brains of adult animals via stereotaxic injection of viral expression vectors. Such application may limit mosaic and off-target effects to the injected brain region, and more importantly, will enable us to examine brain regional effects of mutant genes. Also, by comparing animals at different ages, age-dependent effects of mutant genes can be assessed. Such studies may be particularly useful for large animals to mimic distinct and age-dependent neurodegeneration, which is a pathological feature in different types of neurodegenerative diseases. Also, direct administration of gRNA/Cas9 into specific brain regions of adult large animals to modify neuronal genes does not involve genetic manipulation in germline cells and the extended time for animal development and maturation, which could be an alternative method to rapidly generate large animal models of neurodegenerative diseases.

It should also be pointed out that analysis of the phenotypes of large animal models requires further development of behavioral assays that can be applied to large animals. Also, the number of large animals generated with genetic modification is often not sufficient to provide rigorous statistical analyses. All these obstacles remain to be overcome so that large animal models can be more widely utilized.

## Conclusions

**Fig. 2:**
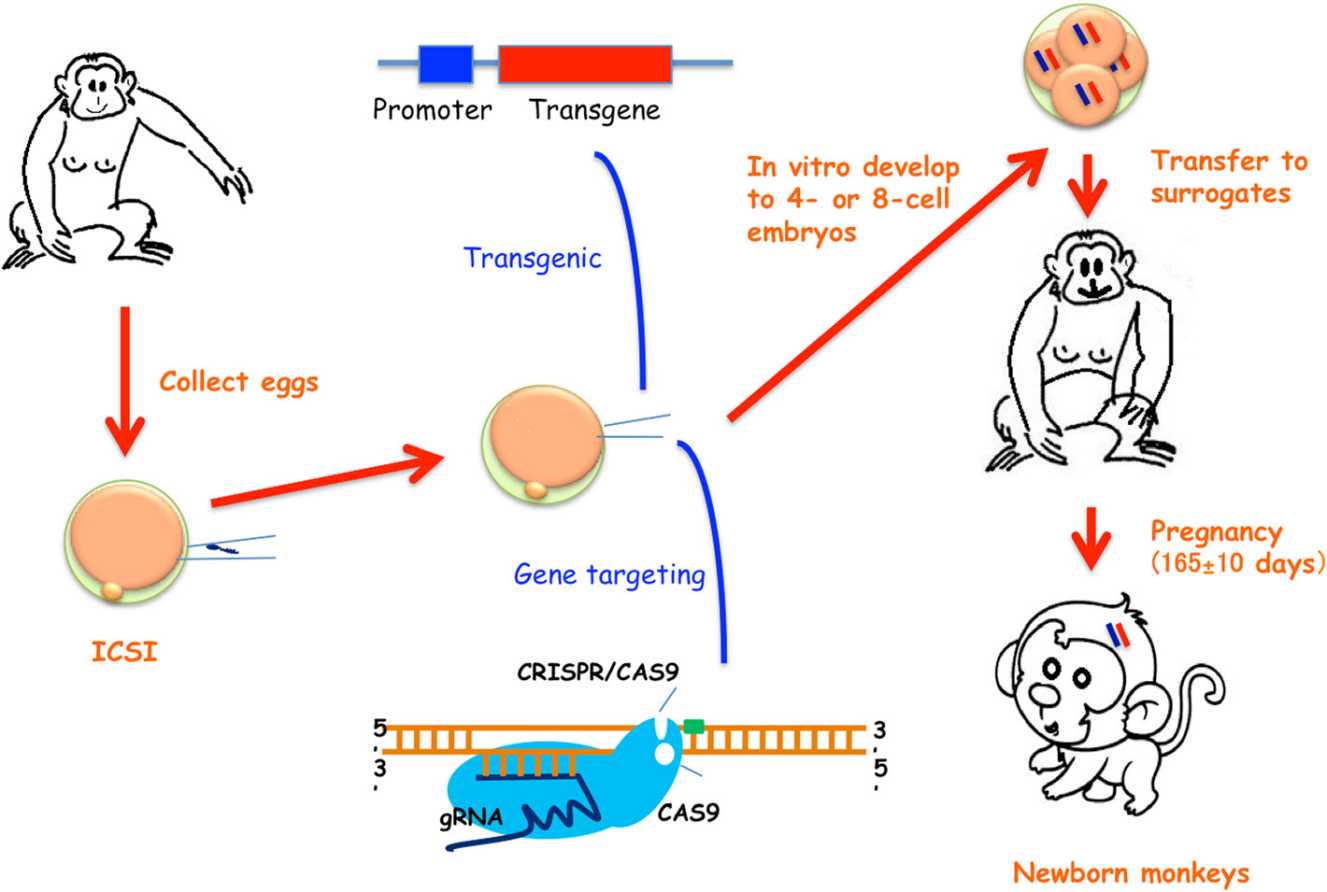
Establishment of non-human primate models of neurodegenerative diseases. In non-human primates, female monkeys are superovulated for collection of eggs, which are subject to intracytoplasmic sperm injection (ICSI) for in vitro fertilization. The fertilized eggs are injected with either lentiviral vectors into perivitelline space to express exogenous transgenes or gRNAs/Cas9 into cytoplasm to target the endogenous genes. The injected eggs then developed to 4- or 8-cell embryos in vitro before being transferred to the surrogate monkeys. After full-term gestational development, the newborn monkeys are examined to verify the presence of transgenes of mutations in the targeted DNAs, which are known to cause neurodegenerative diseases in humans

Transgenic approach and CRISPR/Cas9 can be used to generate large animal models of diseases, such as non-human primate models of neurodegenerative disease (Fig. 2). CRISPR/Cas9 is a new genome modification tool that can efficiently and readily target any gene in the genome in germline cells and somatic cells of different species. Thus, CRISPR/Cas9 makes it possible to implement genome editing in non-human primates and large animals to generate genetic mutations that can faithfully mimic pathology in human patients. Because of the ability of CRISPR/Cas9 to disrupt two alleles, this system can make it possible for founder animals to be investigated for loss of function of the targeted gene. Off-target and mosaic mutations need to be considered when using CRISPR/Cas9. Despite great advances in genome editing, the knock-in rate via CRISPR/Cas9 is still low with current technology. However, new tools for genome editing are being developed quickly and will significantly improve the targeting rate and reduce off-target and mosaic mutation effects. The newly developed CRISPR/Ca9 technology will promote the generation of non-human primates and large animal models of neurodegenerative diseases and enhance our understanding of the pathogenesis of these important diseases.
